# Supplementary material for: Structural intermediates observed only in intact Escherichia coli indicate a mechanism for TonB-dependent transport
Source: eLife. 2021 Jul 12;10:e68548. doi: 10.7554/eLife.68548 (PMC8341980; doi:10.7554/eLife.68548)
Supplement: Supplementary file 1. [file elife-68548-supp1.docx]

|  | component 1 | | | component 2 | | |
| --- | --- | --- | --- | --- | --- | --- |
| [vitamin B_12_] (µM) | **position (Å)** | **width (Å)** | **area (%)** | **position (Å)** | **width (Å)** | **area (%)** |
| 0 | 24.26 | 6.08 ± 1.65 | 40.82 ± 4.08 | 31.16 | 3.52 ± 0.37 | 59.18 ± 3.06 |
| 5 | 24.26 | 6.53 ± 1.39 | 42.86 ± 4.08 | 31.16 | 4.35 ± 0.39 | 57.14 ± 3.06 |
| 10 | 24.26 | 5.17 ± 1.21 | 49.49 ± 4.04 | 31.16 | 4.42 ± 0.61 | 50.51 ± 3.03 |
| 20 | 24.26 | 4.82 ± 1.28 | 64.04 ± 5.62 | 31.16 | 4.13 ± 1.17 | 35.96 ± 4.49 |
| 30 | 24.26 | 5.71 ± 1.16 | 68.04 ± 5.15 | 31.16 | 3.33 ± 0.82 | 31.96 ± 3.09 |
| 60 | 24.26 | 5.03 ± 0.82 | 66.32 ± 4.21 | 31.16 | 4.59 ± 0.9 | 33.68 ± 3.16 |
| 100 | 24.26 | 4.98 ± 0.54 | 68.69 ± 3.03 | 31.16 | 4.5 ± 0.67 | 31.31 ± 2.02 |
