# Supplementary material for: Structural intermediates observed only in intact Escherichia coli indicate a mechanism for TonB-dependent transport
Source: eLife. 2021 Jul 12;10:e68548. doi: 10.7554/eLife.68548 (PMC8341980; doi:10.7554/eLife.68548)
Supplement: Transparent reporting form [file elife-68548-transrepform.docx]

***eLife’s* transparent reporting form**

We encourage authors to provide detailed information *within their submission* to facilitate the interpretation and replication of experiments. Authors can upload supporting documentation to indicate the use of appropriate reporting guidelines for health-related research (see [EQUATOR Network](http://www.equator-network.org/%20)), life science research (see the [BioSharing Information Resource](https://biosharing.org/)), or the [ARRIVE guidelines](http://www.plosbiology.org/article/info:doi/10.1371/journal.pbio.1000412) for reporting work involving animal research. Where applicable, authors should refer to any relevant reporting standards documents in this form.

If you have any questions, please consult our Journal Policies and/or contact us: [editorial@elifesciences.org](mailto:editorial@elifesciences.org).

**Sample-size estimation**

- You should state whether an appropriate sample size was computed when the study was being designed
- You should state the statistical method of sample size computation and any required assumptions
- If no explicit power analysis was used, you should describe how you decided what sample (replicate) size (number) to use

Please outline where this information can be found within the submission (e.g., sections or figure legends), or explain why this information doesn’t apply to your submission:

An appropriate sample size was not computed in these experiments. Rather, complete experiments were repeated, and DEER data recorded until dipolar evolutions were of sufficient echo times and modulation depths reached approximately 4% or better. Modulation depths are relatively low for these labeled cell samples, and this is likely because cells are actively metabolizing during the labeling and washing periods for sample preparation. This information is provided in Methods.

**Replicates**

- You should report how often each experiment was performed
- You should include a definition of biological versus technical replication
- The data obtained should be provided and sufficient information should be provided to indicate the number of independent biological and/or technical replicates
- If you encountered any outliers, you should describe how these were handled
- Criteria for exclusion/inclusion of data should be clearly stated
- High-throughput sequence data should be uploaded before submission, with a private link for reviewers provided (these are available from both GEO and ArrayExpress)

Please outline where this information can be found within the submission (e.g., sections or figure legends), or explain why this information doesn’t apply to your submission:

The EPR spectra and DEER data reported here were repeated anywhere from 1 to 12 times. These represent biological repeats where samples were re-grown and re-labeled for data recording. Most of our repeated samples occurred early in this work as we attempted to optimize labeling conditions. When labeling levels were good, S/N on single 20 second continuous wave EPR scans were 7 to 10 and DEER data reached modulation depths of 4% or greater. Under these conditions, the data were found to be highly reproducible. Cell samples that did not adequately label and DEER runs where power fluctuations resulted in artifacts in the dipolar evolution were discarded. This information is included in Methods.

**Statistical reporting**

- Statistical analysis methods should be described and justified
- Raw data should be presented in figures whenever informative to do so (typically when N per group is less than 10)
- For each experiment, you should identify the statistical tests used, exact values of N, definitions of center, methods of multiple test correction, and dispersion and precision measures (e.g., mean, median, SD, SEM, confidence intervals; and, for the major substantive results, a measure of effect size (e.g., Pearson's r, Cohen's d)
- Report exact p-values wherever possible alongside the summary statistics and 95% confidence intervals. These should be reported for all key questions and not only when the p-value is less than 0.05.

Please outline where this information can be found within the submission (e.g., sections or figure legends), or explain why this information doesn’t apply to your submission:

Error bands in the distance probability distributions obtained by DEER are provided in the Manuscript Figures and Figure Supplements. This error analysis is incorporated into the LabVIEW program LongDistances and utilized 100 variations at the default values for background noise, start time, dimensionality, and regularization error. For model-based fitting LongDistances provides error estimates for distance components in the fit. Supplementary File 1 contains this error analysis for the model-based fitting used in Figure 2D. The DeerNet routine in DeerAnalysis was used to process the data in Figure 3 – figure supplement 2. This also provides an error range that is plotted in this figure. This information is provided in Methods under “Data Processing.”

(For large datasets, or papers with a very large number of statistical tests, you may upload a single table file with tests, Ns, etc., with reference to sections in the manuscript.)

**Group allocation**

- Indicate how samples were allocated into experimental groups (in the case of clinical studies, please specify allocation to treatment method); if randomization was used, please also state if restricted randomization was applied
- Indicate if masking was used during group allocation, data collection and/or data analysis

Please outline where this information can be found within the submission (e.g., sections or figure legends), or explain why this information doesn’t apply to your submission:

No group allocation was carried out. As a result of the way the samples were prepared and measured, some but not all the data acquisition was blinded.

**Additional data files (“source data”)**

- We encourage you to upload relevant additional data files, such as numerical data that are represented as a graph in a figure, or as a summary table
- Where provided, these should be in the most useful format, and they can be uploaded as “Source data” files linked to a main figure or table
- Include model definition files including the full list of parameters used
- Include code used for data analysis (e.g., R, MatLab)
- Avoid stating that data files are “available upon request”

Please indicate the figures or tables for which source data files have been provided:

A data folder containing Source Data (the raw unprocessed DEER data) from the Bruker Elexsys E580 has been made available in the format generated by the Bruker Xepr software (*.DSC, *.DTA). A Pymol session file is also provided that includes 300 structures that are consistent with the distributions obtained by DEER for V90R1-T188R1-R14A and V90R1-S237R1-R14A in the presence of vitamin B12.
